# Differing effects of age and starvation on reproductive performance in *Drosophila melanogaster*

**DOI:** 10.1038/s41598-019-38843-w

**Published:** 2019-02-15

**Authors:** Emily R. Churchill, Calvin Dytham, Michael D. F. Thom

**Affiliations:** 1 Department of Biology, University of York, York, YO10 5DD UK; 2 School of Biological and Marine Sciences, University of Plymouth, Plymouth, PL4 8AA UK

## Abstract

Successful reproduction requires the completion of many, often condition-dependent, stages, from mate searching and courtship through to sperm transfer, fertilisation and offspring production. Animals can plastically adjust their investment in each stage according to the physical and social environment, their own condition, their future reproductive potential, and the condition of their partner. Here we manipulate age and condition, through a nutritional challenge early or late in life, of both male and female *Drosophila melanogaster* and measure the effects on courtship, mating, and fitness when paired with a standardized (unmanipulated) partner. Older males were slower to start courting and mating, and courted at a slower rate, but males were indifferent to female age or condition despite older females laying and hatching fewer eggs. Female condition had a substantial effect on mating acceptance rate, which dropped dramatically after starvation, and particularly recent starvation experience. In contrast, male condition had little effect on any of the components of reproductive performance we measured. Intriguingly, we found no evidence for additive or multiplicative effects of ageing and starvation: the only significant interaction between these variables was on male latency to initiate courtship – older males were slower to start courting unless they had experienced starvation early in life. These results indicate that the immediate costs of mating differ between males and females, and that the sexes differ in their perception of the opportunity cost sustained by refusing a mating opportunity. Our results support the idea that ageing has more wide-ranging impact on reproductive behaviours than does nutritional challenge.

## Introduction

Among animals, reproduction comprises a sequence of events or stages that incur costs for both sexes. In many species males pay costs associated with mate searching, courtship, copulation, and in the production and transfer of ejaculates. For females, copulation is also costly, and there are energetic and other costs associated with offspring production and oviposition or nest site selection. Although all stages need to be completed to achieve successful reproduction, individuals vary their investment in the individual components in response to their own condition^1^ and the social environment^2^.

An individual’s own quality is partly determined by genotype, and may be compromised by senescence^3^ or limited access to resources^4^. Ageing and nutritional challenge are expected to lead to an overall reduction in reproductive capacity, although this effect is not necessarily the consequence of a decline across all stages of reproduction. For example, nutrient restriction in female earwigs *Forficula auricularia* reduced clutch size and hatching success rather than mating rate or acceptance rate^4^, while a high protein diet in male crickets *Teleogryllus commodus* led to an increase in courtship rate^5^. Indeed, while ageing and starvation limit the total future reproductive capacity of an individual, they may initially increase reproductive investment in response to these stresses to compensate for low future opportunities for reproduction^4^. This phenomenon is termed terminal investment^6^, and represents a trade-off of future against current reproductive investment. As mating-related traits are often condition dependent, they may be traded-off against each other not only between but also within reproductive bouts. For example, older male *Drosophila melanogaster* decrease mating rate but increase mating duration^7^, with the latter perhaps compensating for the effects of the former. However, trade-offs may not always be possible, and individuals of lower quality may be constrained to the extent that they cannot compensate^8^.

Alongside the effects of ageing, condition and food availability, the social environment also affects how much and where individuals invest in reproduction, particularly the quality and availability of mating partners. On meeting a partner of relatively low quality an individual can respond by either reducing reproductive investment in anticipation of relatively poor resulting fitness, or increasing investment to compensate for partner quality (differential allocation vs reproductive compensation respectively^9^).

Here we investigate the combined effects of age and nutrition on the reproductive behaviour and fitness of male and female *Drosophila melanogaster*. Age and starvation are both known to have substantial effects on male reproduction in this species when tested independently. Males aged 6 weeks take longer to mate and mate for a longer duration than those aged 1 week^7^. Females mated to these males were also less likely to produce eggs and had lower overall reproductive success, and, unsurprisingly given their lower returns from mating with older flies, there was also evidence of female preference for younger partners^7^. Low protein diets similarly reduce male siring success when the male was second to mate (though not when he was first), although there were no effects on mating duration or latency^10^, and males on the lowest nutrient diets were least-preferred by females^10^. Ageing and starvation effects appear to be less pronounced, or less well-studied, in female *Drosophila*. However, male *Drosophila* are responsive to female age, at least in some circumstances, as young female *D. melanogaster* are preferred by sperm-depleted males, but not by untreated males^11^, and young females receive more sperm than older females^12^. Female receptivity, though, does not appear to be affected by their own nutritional status or age^11^.

While there is good evidence that both ageing and nutritional challenge can affect reproductive tactics in a number of invertebrates including *Drosophila*, previous studies have focussed on the effects of these two challenges in isolation^7,10,13–18^ or more rarely, the combined effects in a single sex^19,20^. Here we focus on the combined effect of ageing and nutrition in both sexes, since in the natural environment the costs must often co-occur – a food shortage caused by a weather event, for example, is likely to affect all adult age classes – and be experienced by both sexes. Exposure to more than one cost simultaneously may lead to previously unidentified additive or multiplicative effects. By simultaneously manipulating both age and acute starvation stress in both male and female *D. melanogaster* we aimed to quantify the effects of the two challenges when experienced simultaneously. Furthermore, we expected that different patterns of food restriction would cause different compensatory responses. Contemporary nutrient deficiency causes a loss in condition, reducing the range or quantity of energetically demanding responses that an individual can display in response to an additional challenge. Early-life nutrient restriction may instead lead to long term changes in behaviour and physiology as the individual is ‘primed’ for a high risk of future challenges. While ageing unavoidably reduces future reproductive performance, individuals may alter their reproductive strategy in response. Contemporary nutritional restriction could prevent these compensatory responses by reducing an individual’s capacity to alter behaviours such as courtship rate or oviposition, while early-life nutrient restriction may instead cause a shift in an individual’s reproductive schedule (in the expectation of poor future conditions) and the effects of ageing may therefore be experienced differently.

Finally, condition and age affect not only an individual’s behaviour and physiology but also its probability of being selected as a mate^7,11,15^, and indeed the resulting behaviour and physiology of its partner^10^ if mating is successful. We therefore tested the interacting effects of age and nutritional restriction separately in both the chooser and the prospective mate.

## Results

### Courtship

**Figure 1:**
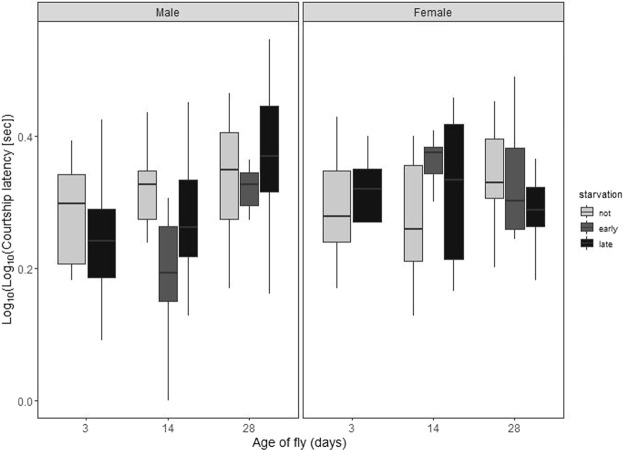
The effect of fly age and starvation status on latency to start courtship (doubly logged for normality). Boxes span the 25^th^ to 75^th^ centiles, the centre line is the median, and whiskers show the most extreme data point. Courtship latency significantly declined in older males, but this was counteracted in early starved males which initiated courtship more rapidly at ages 14 and 28 days. Neither the starvation nor age status of the female influenced male latency to initiate courtship.

Among males that showed courtship behaviour within 30 minutes (96.9% of pairs), the oldest individuals were significantly slower to initiate courtship (double-log transformed; F_2,104_ = 10.91, p ≪ 0.001; Fig. 1). This effect was counteracted in males which had experienced early-life starvation: these individuals started courting sooner than unstarved or recently-starved males at all ages (F_2,104_ = 4.95, p = 0.009; starvation:age interaction F_3,101_ = 2.01, p = 0.117; Fig. 1). In contrast, neither age nor starvation status of the female affected the speed at which a male started to court her (both F_2,101_ <2.54, p > 0.084; Fig. 1).

**Figure 2:**
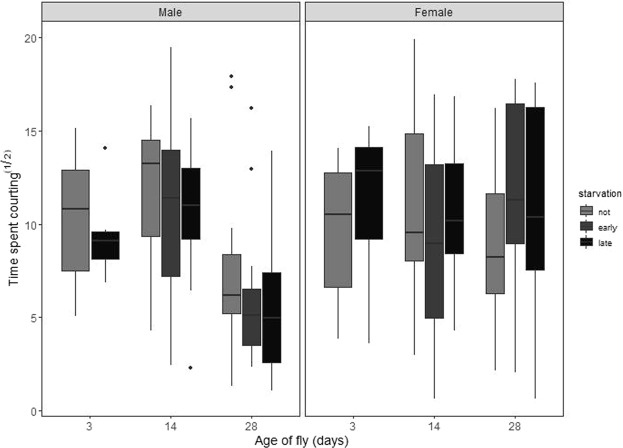
The effect of fly age and starvation status on the amount of time a male spent courting in his first 5 minutes of courtship (square-root transformed). Graphical components as for Fig. 1; points represent outliers more than 1.5x the 25^th^ to 75^th^ centile range. Male courtship effort declined with age, but was unaffected by starvation. Neither the starvation nor the age status of the female affected how much courtship a male directed towards her.

Courtship intensity declined markedly with male age (square-root transformed; F_2,99_ = 16.62, p ≪ 0.001; Fig. 2) but not in response to male starvation status (F_2,99_ = 1.47, p = 0.235). Like courtship latency, courtship intensity was not affected by either female age or starvation status (both F_2,91_ <0.38, p > 0.683; Fig. 2).

### Copulation success

**Figure 3:**
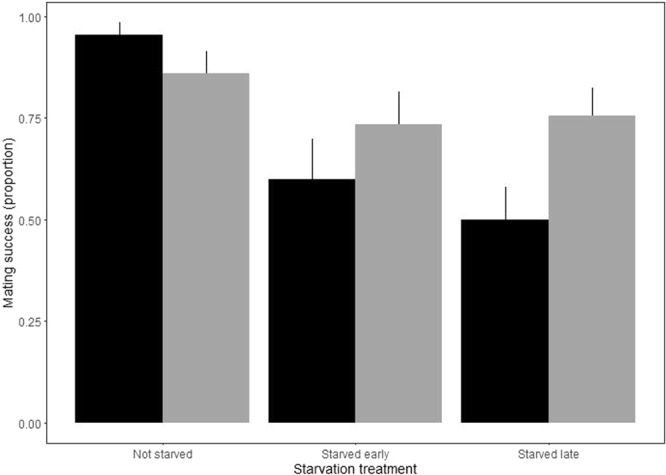
The proportion of paired flies in which copulation occurred. Black bars represent pairs in which the female was starved, grey bars the pairs in which the male was starved; all age treatments were pooled for this analysis (age did not significantly affect probability of copulation). Errors are standard errors of proportions $\sqrt{\frac{p\times (1-p)}{n}}$. Females were significantly less likely to copulate if they were starved, whether early or late in life. There was no statistically significantly equivalent effect of male starvation treatment.

Female starvation status had a striking effect on whether or not courtship then led to successful copulation. Non-starved females copulated 95% of the time, compared to 70% in early-starved and 41% in recently-starved females (GLM with binomial errors; deviance 30.1, df = 94, p ≪ 0.001; Fig. 3). Females were not courted less when they were starved (see above), and including courtship in the statistical model of copulation probability did not change the outcome. There was no evidence that females rejected males based on male starvation status (deviance 0.51, df = 101, p = 0.774).

Unlike starvation, age had no significant effect on copulation success of either males or females (both deviance <1.46, both p > 0.482). Contrary to expectations, a male’s intensity of courtship did not significantly influence whether or not mating occurred (focal males: deviance = 2.52, df = 1,94, p = 0.112; focal females: deviance = 0.002, df = 1,85, p = 0.962).

### Female rejection behaviour

**Figure 4:**
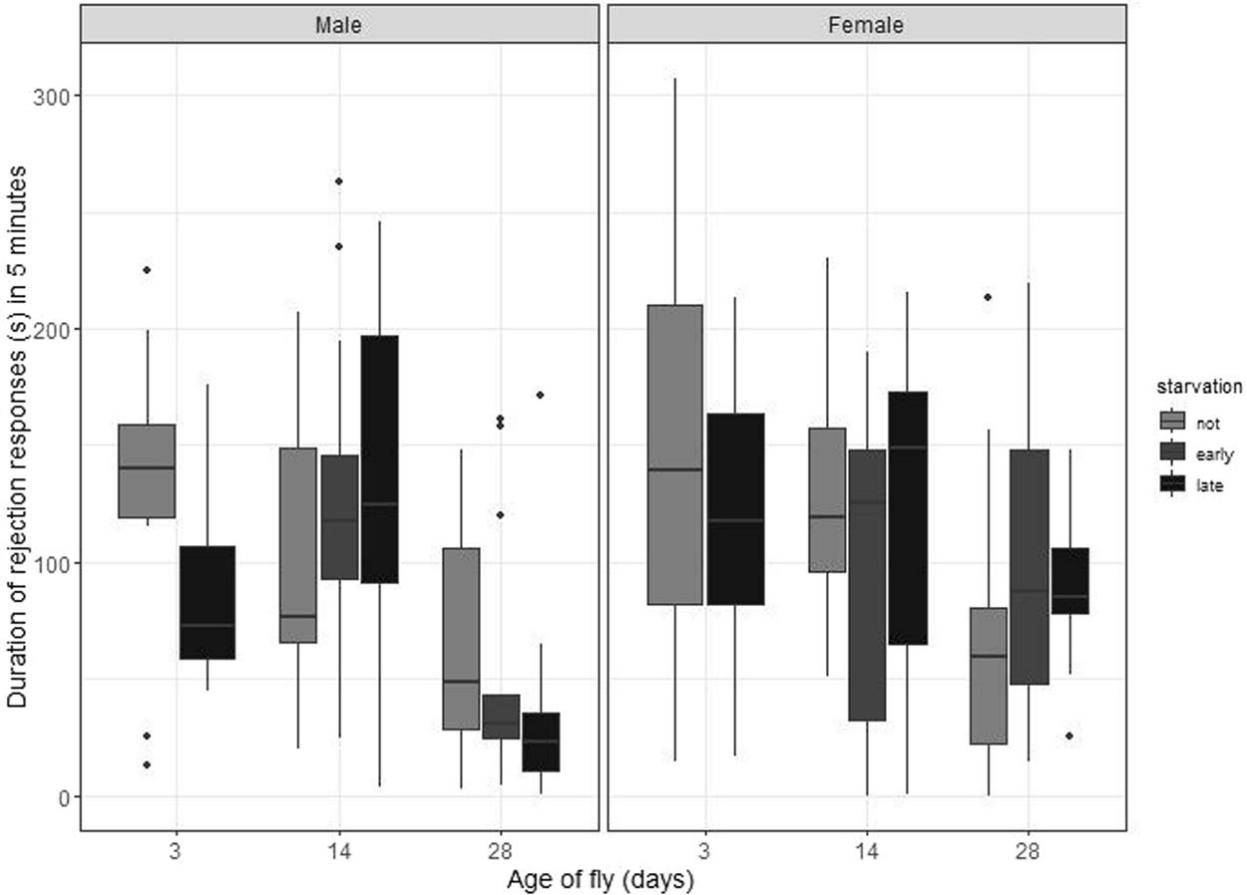
The amount of time females spent directing rejection behaviour towards males, in the first 5 minutes of courtship. Graphical parameters as for Figs 1 and 2. Older males were less likely to receive rejection behaviours, as were starved males (though the effect of starvation was not detected at the intermediate male age of 14 days). Female age and starvation did not significantly affect the level of rejection effort she displayed.

A pair may fail to mate after courtship is initiated for a number of reasons, among them persistent rejection of the male by the courted female. The female’s condition did not affect the amount of rejection behaviour she displayed (starvation and age: F_2,94_ <2.35, p > 0.101). However, the male’s condition did: young starved males, and all older males irrespective of starvation status, received relatively few rejections (starvation*age F_3,97_ = 3.08, p = 0.031; main effect of age: F_2,97_ = 22.39, p ≪ 0.001; Fig. 4). This was not because these males spent less time courting: although there was a significant positive association between time spent courting and duration of rejection behaviours received (F_1,95_ = 22.80, p ≪ 0.001) male condition remained significant in the model (starvation*age F_3,95_ = 3.70, p = 0.014; age F_2,95_ = 27.93, p ≪ 0.001). Counterintuitively, males that were successful in obtaining a mating received significantly more rejection behaviour from females (focal males receiving rejection: mating success deviance = 13.16, df = 1,95, p < 0.001; focal females performing rejection: mating success deviance = 7.46, df = 1,88, p = 0.006).

### Copulation latency

While females showed less rejection behaviour towards older males, these older males nevertheless took longer to start copulating after courtship began (F_2,78_ = 5.66, p = 0.005). Starvation had no equivalent effect on a male’s latency to begin copulating (F_2,78_ = 2.75, p = 0.07). Despite females being less likely to copulate when they were starved, among those females that did mate the latency was unaffected by starvation (or by age; both F_2,69_ < 0.725, p > 0.488). Males’ courtship intensity in the first 5 minutes did not significantly influence how quickly copulation started (focal males F_1,78_ = 0.45, p = 0.503; focal females F_1,62_ = 3.67, p = 0.06).

### Mating duration

A weak interaction between age and starvation status affected how long males mated for (F_3,79_ = 3.07, p = 0.033) due to a trend towards longer matings in young, starved flies. Neither of the main effects was significant in this model (age: F_2,79_ = 1.055, p = 0.353; starvation: F_2,79_ = 0.452, p = 0.638), and there were no significant effects of female treatment on mating duration (all F <0.848, all p > 0.4331).

### Fitness

Among pairs that mated, total number of eggs laid over 48 h significantly declined with increasing age in females (GLM with quasi-Poisson errors; deviance = 151.4, p = 0.004) but not males (deviance = 57.0, p = 0.246). Starvation was not a significant predictor in either model (deviance < 56.2, p > 0.251). Among pairs that mated, increasing age of both sexes significantly decreased the proportion of eggs hatched (female age: F_2,49_ = 5.17, p = 0.009; male age: F_2,54_ = 4.8, p = 0.012).

## Discussion

We found the effects of ageing on male mating performance to be pronounced. There were particularly substantial effects of male age on courtship: older males were slower to start courting, performed less courtship when they began, and then took longer to start mating. This increased delay between the initiation of courtship and female acceptance we interpret as a reduction in female motivation to mate with these older males. Reduction in courtship latency and intensity with age has been reported in other insects^21,22^, though not to our knowledge in *D. melanogaster*. There are previous reports of mating latency being affected by male age in *D. melanogaster*^7^, though our results did not support that study’s finding that mating duration increased in the oldest males, possibly due to the difference in age between our oldest males (4 weeks) and theirs (6 weeks^7^). Nevertheless, our results match expectations of the effects of senescence: decreasing performance, including in reproduction, and increased probability of death with increasing age. There was one exception to these trends, namely that males starved early in life initiated courtship more rapidly than did males in the other treatments. This could be a terminal investment response^6^ – these males were less than two days from death by starvation – though terminal investment is notoriously difficult to demonstrate conclusively, and there is no other evidence for such a strategy among the behaviours recorded here. However, the effect of starvation on courtship latency was substantial, and starvation appears to impart a real urgency to court among males that is strong enough to override the negative effects of ageing.

Despite older males experiencing a decline in courtship performance, females were still willing to mate with them – albeit taking longer to do so – because the probability of successful copulation was not significantly influenced by male age. These males were a relatively poor investment, however, siring a lower proportion of hatching eggs than did younger males. Intriguingly, age had no detectable effect on mating success in either sex, despite this being the only measured variable that significantly affected fitness.

We assessed whether male mating success could be explained by the number of rejections a female responded with, and found that older males received fewer rejections than did young males. One possible explanation for this is that older males are preferred by females, as they are in *D. subobscura*^15^, so that while older males are slower at initiating and performing courtship, females are less likely to reject their advances. However, we saw no other evidence for preference for older males, and unlike the monogamous *D. subobscura*, our study species is promiscuous, perhaps reducing the benefits of mating with older males. An alternative explanation for the reduced rejection behaviour towards older males may be that these males have reduced vigour, giving them higher motivation to avoid persistent courtship with unreceptive females. It appears plausible that females match their level of rejection behaviour to the courtship effort shown by the male, and that older males are weaker and easier to rebuff. Thus we suggest that older males court less but more strategically, requiring less rejection behaviour by females and leading to an equal acceptance rate to the more persistent but less targeted efforts of younger males.

Notwithstanding the hypothesis proposed above, the significance of rejection behaviour by females is currently difficult to interpret, and would benefit from further investigation particularly as this behaviour is considerably less well studied than the behaviour of males during courtship^23^. However, the hypothesis that older males take less effort to reject is consistent with our observation that starved males also received fewer rejection behaviours by females, and it also aligns with the strong positive association we observed between duration of courtship and the quantity of rejection behaviour received. The idea that females match their rejection effort to the level of courtship they are receiving, perhaps testing males’ commitment or vigour, may also underlie the surprising observation that successful matings follow a higher frequency of rejection behaviours than do unsuccessful matings. We cannot currently explain the observation that male courtship effort has no discernible effect on his probability of obtaining a successful mating. One explanation may be that the males in our study were naïve, and there is evidence that courtship performance improves with experience^24^. Alternatively, our measure of courtship effort (the amount of time spent courting in the first 5 minutes of courtship) may not accurately reflect the qualities in which females are interested^23^.

Males showed no sensitivity to the quality of potential mating partner in any of the measured parameters; they courted at the same rate irrespective of female age or starvation status. This result is surprising, since courtship is expensive to males in this species^25^, and there would appear to be little value in courting low quality females or those at the end of their life. Previous authors have found male preference for young and/or well-nourished females, though this was only the case for sperm-limited males^11^. Our result may thus be a consequence of our use of standardized, 7 day old, non-starved, males, which suffer no substantial consequences, including relatively little opportunity cost, for courting each female they meet. Indeed, in this design the focal female was the first female this male had encountered, so it may be that the benefits of achieving at least some reproductive success outweighed the risks of mating with a poor-quality female.

While age had clear detrimental effects on male courtship behaviour, neither sex showed any effect of age on receptivity to mating. In another dipteran, the medfly, *Ceratitis capitata*, ageing significantly increases female receptivity and older females are more likely to mate than younger ones^20^. Those authors also found good nutrition similarly improved female receptivity, and in our study only this variable affected how likely a female (but not a male) was to mate: the more recently she had experienced a period of starvation, the less likely that mating would occur. This pattern would suggest that females respond to their weakened state by reducing willingness to copulate. Mating is costly to female *D. melanogaster*, at least partly as a consequence of the toxic accessory proteins transferred by males during mating^26^ but probably also energetically, and this may be an additional burden they cannot afford during periods of nutrient restriction. Males, by contrast, continue to court females even when old and starved, following what has been termed the ‘reproduction at any cost’ hypothesis^20,27^.

Surprisingly, our factorial manipulation of age and starvation revealed little evidence of any interaction between our two main stressors, despite each treatment having substantial consequences for some behaviours in at least one sex. The only clear interaction affected male initiation of courtship where the trend for older males to start courting later was reversed in early-starved males. The absence of any further interactions between stressors, particularly the multiplicative effects we were anticipating, remains to be explained. It may be that the treatments were already of sufficient severity that no further increase in response could be stimulated by the addition of a second stressor. A previous study manipulating age and nutritional status in female *D. melanogaster* revealed a very weak interaction effect on reproduction only on one variable: total fitness^11^. Nevertheless, in other species, ageing and nutritional restriction can interact in influencing mating behaviour: for example, Wilgers & Hebets^28^ demonstrated that the effect of restricted diet on mating in female wolf spiders *Rabidosa rabida* was mediated by age, with younger females being less likely to mate on a restricted diet than were old females. Similarly there is evidence of an interaction between age and nutritional status on female receptivity in the medfly *Ceratitis capitata*^20^.

Finally, we set out to explore the manipulation of individual quality by two quite different mechanisms: early life starvation and recent starvation. Early life starvation was intended to get individuals off to a relatively slow start (the inverse of the silver spoon effect^8^), and also institute a memory of poor environmental conditions which we expected might influence mating investment decisions later in life. Recent starvation was designed to ensure individuals were stressed at the time of reproduction. As it turned out, neither had particularly widespread effects on the variables measured here, though early life starvation led to more rapid onset of courtship in males, and any exposure to starvation led to reduced egg production in females. It seems likely that even brief early life starvation causes a reduction in female egg production capacity: nutritional restriction reduces the number of viable egg chambers, and hence normally-developing eggs, in female *Drosophila*^29^. It may be that these effects persist long after normal nutrition is resumed. In other species, early life effects are known to have profound effects on subsequent development^8^, influencing behavioural and physiological performance including e.g. egg production in birds^30^.

We have, for the first time in this species, tested the simultaneous effects of starvation and ageing stress on reproduction variables in both sexes. There were clear sex-specific effects, with age affecting males more than females, and starvation having some influence on performance in both sexes. Both sexes maintained performance in most variables while suffering a decline in others in response to stress, suggesting either that different components of mating are unequally susceptible to condition in the two sexes, or that remaining resources are strategically targeted at the most essential functions, representing an instantaneous trade-off between behaviours affecting fitness.

## Methods

We used an F1 cross of the Oregon R and Canton S strains of *Drosophila melanogaster* to produce a relatively outbred, heterozygous experimental stock. Each base strain was maintained in 40 ml vials containing 7 ml of standard agar-based medium (40 g brewers’ yeast, 40 g sucrose, 14 g agar, 10 ml methyl-4-hydroxybenzoate per L) at a constant temperature of 25 °C in a 12 L:12D cycle. Approximately 25 flies were housed per vial to minimise density effects, and adults from all vials of each strain were mixed every four days. F1s were produced by pairing four OR females with four CS male in a standard vial and removing the adults after three days.

To test whether the reproductive success was affected by the interaction between starvation (three levels) and age (three levels), in a fully factorial design with one exception, noted below. The three levels of the age treatment were 3, 14, and 28 days (flies of the parent strains live for an average of 40–60 days in captivity^31,32^); the three starvation treatments were: not starved, starved on days 1 & 2 (early starved), or starved on the final two days before testing (late starved), i.e. on days 12 and 13 or days 26 and 27. The 3 day old flies could not be starved in both patterns due to their young experimental age – they were starved only for the last two days before testing. This resulted in eight starvation x age treatment groups. We tested the effects of these variables in both sexes, but to simplify interpretation we manipulated age and nutrition in one sex at a time, always pairing the treatment sex with a standardized mating partner (opposite sex, 7 days old, maintained on standard nutrient agar). Pilot data demonstrated that the mean survival period of N = 37 flies under starvation conditions was 3.7 ± 1.3 (SE) days, so a 2 day starvation period was chosen to be energetically taxing but not fatal.

At the end of the treatment period, flies were paired in a ~1 ml cylindrical well of a 9-well mating arena; each well contained approximately 0.01 g of brewer’s yeast granules. The arena was set on a hot plate set to 25 °C to maintain constant internal temperature. Females were placed in the arena first, a clear plastic sheet was introduced in the horizontal plane through the arena, and the male introduced on top of this before the whole apparatus was sealed with a final clear plastic sheet. The pair were kept separated in this way for five minutes to allow them to acclimatise before the separating sheet was removed and the pairs allowed to interact. They remained in the arena for 105 minutes. Behaviour was recorded using a Logitech HD Pro Webcam C910 1920 × 1080 pixel camera connected to a computer, and subsequently transcribed from video files using custom-written event recording software. We measured seven variables to quantify components of courtship and reproduction. These were: courtship latency (in s; time from introduction of the fly to the arena and first observed courtship event); courtship intensity (in s; time spent courting in the first 5 minutes of courtship, scaled accordingly if the fly mated in that time); female rejection intensity (in s; total time the female spent fleeing or kicking at males, scaled as for courtship intensity); mating latency (in s; time between initiation of courtship and start of mating); mating duration (in s); number of eggs laid (over 48 h, sum of two 24 h laying periods); and hatching success (%, measured 48 h after laying). Courtship frequency (number of wing raises to 90°) per minute in the first 5 minutes of courtship is strongly correlated with courtship frequency per minute until mating is initiated (r = 0.634, T_28_ = 4.33, p < 0.001; unpublished data). While we chose to use courtship duration over courtship frequency here, we measured both variables and found them to be closely correlated (r = 0.65, T_198_ = 12.1, p ≪ 0.001). We also measured male chasing, tapping, licking and mounting attempts: the frequency of each of these measures was also correlated with courtship intensity (all r > 0.16, all p < 0.022).

After the 105 minute mating opportunity, or after copulation had ceased, we measured female egg production and subsequent hatching success. Each female was aspirated into an empty 40 ml vial, which was inverted onto a disc of standard agar medium, surrounded by damp sand for moisture retention. Females were moved to a second disc of agar after 24 h, and eggs counted at 24 h and larvae at 48 h to allow egg production and hatching success to be quantified.

We started with N ≈ 20 flies per age/sex/starvation treatment combination, but final sample sizes ranged between 12 and 19 per treatment combination, as a result of, for example, failure to show courtship or failure to mate. We analysed the effects of starvation and ageing on reproductive variables in each sex separately. Although we had all age treatments for both ‘non starved’ and ‘late starved’ flies, there was no three day old treatment of ‘early starved’ flies resulting in an incomplete factorial design. Both age and starvation treatment, and their interaction, were included in statistical models – when interpreting the interaction term recall that one factor combination out of 9 (three day old|early starved) is missing. We started with the maximal model including interaction terms, and then excluded terms by stepwise elimination until only significant terms and both main effects (age and starvation) remained. Where additional explanatory covariates were included in the model these are noted in the results. For continuous response variables we used linear models with appropriate transformations to ensure normality; mating success was analysed using a generalized linear model with binomial errors and egg production a GLM with quasi-Poisson error distribution to correct for overdispersion. All analyses were performed in R version 3.4.1^33^.
